# Synbiotic HQP08 Supplementation for Hair Loss and Depressive Symptoms: An Open-Label Pilot Study of Clinical Outcomes and Gut Microbiome Changes

**DOI:** 10.3390/nu18193111

**Published:** 2026-09-22

**Authors:** Zhilu Xu, Raphaela I. Lau, Haoyu Qi, Martin C. S. Wong, Kingsley H. N. Chan, Ivan S. F. Lau, Jessica Y. L. Ching, Pui Kuan Cheong, Francis K. L. Chan, Siew C. Ng

**Affiliations:** 1 Microbiota I-Center (MagIC), Hong Kong SAR, China; zhiluxu@cuhk.edu.hk (Z.X.); lauhiulamiris@link.cuhk.edu.hk (R.I.L.); haoyuqi@cuhk.edu.hk (H.Q.); fklchan@cuhk.edu.hk (F.K.L.C.); 2 Department of Medicine and Therapeutics, The Chinese University of Hong Kong, Hong Kong SAR, China; sfilau@cuhk.edu.hk (I.S.F.L.); jessicaching@cuhk.edu.hk (J.Y.L.C.); jcheong@cuhk.edu.hk (P.K.C.); 3 Institute of Digestive Disease, State Key Laboratory of Digestive Diseases, Li Ka Shing Institute of Health Science, The Chinese University of Hong Kong, Hong Kong SAR, China; 4 Jockey Club School of Public Health and Primary Care, Faculty of Medicine, The Chinese University of Hong Kong, Hong Kong SAR, China; wong_martin@cuhk.edu.hk; 5 Dermamier Skin and Laser Center, Hong Kong SAR, China; kingsleychanhk@gmail.com; 6 Center for Gut Microbiota Research, Faculty of Medicine, The Chinese University of Hong Kong, Hong Kong SAR, China

**Keywords:** alopecia, hair loss, synbiotic, gut–skin axis, gut microbiome

## Abstract

**Background**: Gut microbiota dysregulation has been implicated in hair loss via the gut–skin axis, but human interventional data are limited. We previously showed that *Bifidobacterium longum* HK003 reverses alopecia-like phenotypes in mice. **Methods**: We conducted an open-label, single-arm pilot study to evaluate an HK003-containing synbiotic (HQP08) in 32 adults with self-reported hair loss over 8 weeks. The primary endpoint was the proportion of participants reporting improvement on the Patient Global Impression of Change (PGI-C) scale at week 8. Secondary outcomes included changes in hair loss severity, 60 s hair count, depressive symptoms (PHQ-9), safety, and gut microbiome composition (Chinese Clinical Trial Registry ChiCTR2500112698). **Results**: At week 8, 62.5% (20/32) of participants reported PGI-C improvement. Significant reductions were observed in hair loss severity (*p* = 0.0008), 60 s hair count (*p* = 0.0048), and PHQ-9 scores (*p* = 0.002). Younger age was associated with PGI-C response (OR per year 0.86, 95% CI: 0.73–0.98, *p* = 0.037). Metagenomic analysis identified engraftment of *B. longum* HK003-like profiles in a subset of participants at week 12. Changes in microbial pathways related to thiamine metabolism, vitamin B12 salvage, and lipopolysaccharide biosynthesis were associated with ΔPHQ-9 (FDR-adjusted *p* < 0.05), but not with hair-related endpoints. No serious adverse events occurred. **Conclusions**: This first-in-human pilot study suggests that HQP08 was well tolerated and was associated with within-participant changes in hair-loss measurements and PHQ-9 scores. Randomized controlled trials are warranted to confirm efficacy and further elucidate mechanisms within the gut–brain–skin axis.

## 1. Introduction

Emerging evidence highlights the critical role of the gut microbiome, extending its influence beyond the gastrointestinal tract to extra-intestinal organs, including the skin [1]. Hair loss, or alopecia, is a common condition with a considerable psychosocial burden [1,2], yet current pharmacological treatments are limited by tolerability concerns and long-term compliance issues [3,4,5,6]. Recent conceptualizations of the gut–skin axis suggest that intestinal dysbiosis may drive systemic inflammation and immune dysregulation, contributing to dermatological and follicular disorders [1,7]. Consequently, targeted manipulation of the intestinal microbiota represents a promising frontier for treating extra-intestinal conditions like alopecia.

Hair follicle cycling is regulated by local epithelial–mesenchymal interactions and systemic immune–metabolic status, and its disruption drives immune-mediated alopecia areata [8,9]. Intestinal dysbiosis has been linked to dysregulated skin immune responses and heightened systemic inflammation, a framework now commonly described as the gut–skin axis [1]. Consistent with this, patients with alopecia areata show gut microbiota alterations, including reduced commensal taxa and enrichment of pro-inflammatory bacterial groups [7]. Notably, incidental clinical observations have reported hair regrowth in patients with alopecia universalis following fecal microbiota transplantation (FMT), suggesting that restoration of gut microbiota may affect hair follicle cycling [10].

Building on these insights, our preclinical work demonstrated that FMT or administration of a defined *Bifidobacterium longum* strain (HK003) reversed severe hair loss in mice, restoring anagen follicle counts and skin thickness [11]. These findings highlight targeted microbial modulation as a potential systemic strategy for hair loss management. Addressing this gap is clinically relevant, as microbiome-directed interventions may offer a non-invasive, well-tolerated approach that targets immune–metabolic pathways implicated in follicle biology.

Here, we report an open-label pilot study evaluating the tolerability and preliminary clinical changes associated with HQP08, a synbiotic formulation containing *B. longum* HK003, in adults with self-reported hair loss. Outcomes included hair loss severity and shedding, psychological well-being, and quality of life, complemented by longitudinal metagenomic profiling to assess gut microbiota shifts and strain-level dynamics.

## 2. Materials and Methods

### 2.1. Study Participants

This was an open-label pilot study conducted at the Prince of Wales Hospital, Hong Kong. Adults aged 18–50 years with self-reported hair loss were eligible if they were literate and able to provide written informed consent. Exclusion criteria included a history of hair transplantation; psoriasis or severe scalp infection; cancer within the preceding 5 years; current use of minoxidil, finasteride, anti-androgens, or corticosteroids; antibiotic use within one month before enrolment; severe allergy to probiotics or lactose; and known pregnancy or lactation. As a pilot study, the protocol targeted 30 evaluable participants, with enrolment of up to 40 participants. No formal efficacy power calculation was performed; the findings were intended to inform the design of a future randomized controlled trial. Participants received HQP08, a synbiotic formula containing 20 billion colony-forming units per sachet of *B. longum* HK003, with fructooligosaccharides (FOSs), galactooligosaccharides (GOSs), pumpkin seed oil powder, and xylitol, once daily for 8 weeks. HQP08 was manufactured and supplied by GenieBiome Limited (Hong Kong SAR, China). The individual quantities of the non-microbial ingredients are proprietary and commercially confidential and are therefore not disclosed. The eight-week intervention was selected as a pragmatic pilot duration, informed partly by previous clinical data [12] suggesting that hair-loss symptoms may change within approximately three months following administration of a related synbiotic formulation. Clinical outcomes were assessed at baseline and after 8 weeks of HQP08 supplementation. A schematic overview of the study design is shown in Figure 1A. This study was approved by the NTEC-CUHK Clinical Research Ethics Committee (Approval code: 2024.703; Date of approval: 23 January 2025). It was registered with the Chinese Clinical Trial Registry (ChiCTR2500112698).

During the study period, the use of minoxidil hairspray, oral finasteride, and probiotics or prebiotics other than the study product was recorded. Compliance was assessed by counting used sachets at weeks 4 and 8. Although subjects were asked to maintain their usual dietary pattern during the trial period, detailed diet information was not recorded.

### 2.2. Outcomes

The primary outcome was the proportion of participants achieving clinical improvement at week 8, defined as a Patient Global Impression of Change (PGI-C) score of 1–3. Participants with week-8 PGI-C scores of 1–3 were classified as PGI-C responders, whereas those with scores > 3 were classified as non-responders. Secondary and exploratory outcomes included changes in Patient Global Impression of Severity (PGI-S), 60 s hair count, Patient Health Questionnaire-9 (PHQ-9), Generalized Anxiety Disorder-7 (GAD-7), and World Health Organization Quality of Life-Brief Version (WHOQOL-BREF). Safety and tolerability were assessed by monitoring adverse events and serious adverse events throughout the study. The clinical outcomes were specified in the ethics-approved protocol, whereas the detailed microbiome and subgroup analyses were exploratory.

### 2.3. Faecal Metagenomic Sequencing and Profiling

Stool samples were collected at baseline and at week 12 (4 weeks post-intervention) to assess post-treatment gut microbiome changes. Samples were immersed in Norgen preservatives (cat. 63700, Norgen Biotek Corp., Thorold, ON, Canada) and stored at −80 °C. All samples were processed in a single batch upon trial completion. Faecal DNA was extracted using the Qiagen PowerSoil Kit according to the manufacturer’s instructions. Sequencing libraries were prepared from extracted DNA using the Illumina DNA Prep (Illumina, San Diego, CA, USA) and sequenced using an Illumina NovaSeq 6000 System(Illumina, CA, USA), yielding an average of 37.5 ± 10.6 million paired-end reads per sample. Raw sequence reads were quality-trimmed using Trimmomatic (v0.40) [13] with default parameters. Host reads were filtered using Bowtie2 v2.2.3 [14,15,16] aligned to the CHM13 human genome database. Taxonomic profiling was performed using MetaPhlAn v4.2.2 [17] with the mpa_vJan25_CHOCOPhlAnSGB_202503 database. Functional profiling was conducted using HUMAnN v3.9 [18].

### 2.4. Bioinformatics Analysis

Low-abundance features were filtered by retaining taxa or pathways with relative abundance >0.01% in at least 20% of samples, followed by centered log-ratio (CLR) transformation. Microbial community differences were assessed using PERMANOVA (adonis2) on Bray–Curtis distances with permutations constrained within participants. Linear mixed models (LMMs) were fitted using the lmer function with CLR-transformed abundance as the outcome, including time point, age, sex, and BMI as fixed effects and a random intercept for each subject. Significance of the time effect was tested using Type III ANOVA. Associations between clinical improvements and microbiota changes were assessed using Spearman correlations of delta values. *p*-values were adjusted for multiple comparisons using the Benjamini–Hochberg (FDR) method with a significance threshold of *p*_adj < 0.05. Due to the exploratory nature of this pilot study, both nominal and adjusted *p*-values are reported.

### 2.5. Strain-Level Analysis

Strain-level profiling was performed using StrainPhlAn v4 [11,19]. Marker genes specific to *B. longum* were extracted from each metagenomic sample, aligned, and concatenated to construct a maximum-likelihood phylogenetic tree (RAxML, GTR+GAMMA model) including the supplemented reference strain HK003. Pairwise phylogenetic distances were calculated from the tree. *B. longum* HK003-like strains were defined by an HK003-centered cophenetic-distance neighbourhood at a conservative radius that admitted no baseline samples; this assignment was corroborated by an independent, reference-anchored marker-SNP distance criterion (≤500 SNPs to the HK003 reference genome, among samples with ≥50,000 compared sites), which yielded identical calls. Samples within this neighbourhood were classified as *B. longum* HK003-like; others were assigned to endogenous clusters via hierarchical clustering (UPGMA) of the distance matrix, with clusters defined by the largest gap in the merge-height distribution. Engraftment of *B. longum* HK003-like strains was defined as the detection of a *B. longum* HK003-like strain at week 12 in a participant with no detectable *B. longum* HK003-like strain at baseline. Visualization was performed using the ggalluvial [20] package 0.12.6 in R.

### 2.6. Statistical Analysis

All statistical analyses and visualizations were conducted in R version 4.5.3 [21]. Key packages included ape 5.8.1 [22] and phangorn 2.12.1 [23] for phylogenetic analysis, vegan 2.7.3 [24] for diversity and PERMANOVA analyses, lme4 2.0.1 for linear mixed-effects models, ggplot2 4.0.2 [25] for data visualization, and ggalluvial 0.12.6 [20] for alluvial plots. Statistical tests were two-sided, and *p* < 0.05 was considered nominally significant unless otherwise specified. No missing-data imputation or intention-to-treat analysis was performed.

## 3. Results

Between 12 March 2025 and 9 July 2025, 35 participants were enrolled. Two were lost to follow-up and one was excluded after initiating the study product before baseline stool collection. A total of 32 participants with available week-8 clinical outcomes were included in the final analysis. Baseline characteristics are summarized in Table 1. Compliance was 96.6% and 91.6% at weeks 4 and 8, respectively, with an overall mean of 95.8% over the 8-week intervention.

### 3.1. Primary Outcome

At week 8, 62.5% (20/32) of participants reported overall improvement, defined as a PGI-C score of 1–3 (Figure 1B). Among these, 9.4% were rated as “much improved” and 53.1% as “minimally improved”. The distribution of PGI-C responses at weeks 4 and 8, together with a sensitivity analysis using a stricter response definition (PGI-C ≤ 2), is shown in Figure S1A,B. Participants classified as responders (PGI-C ≤ 3) at week 8 were significantly younger than non-responders (PGI-C > 3) (Wilcoxon rank-sum test, *p* = 0.023; Figure 1C). In univariable analyses, younger age was associated with PGI-C improvement at week 8 (OR per year 0.86, 95% CI: 0.73–0.98, *p* = 0.037); other baseline characteristics, including baseline PHQ-9, GAD-7, sex, BMI, baseline hair count, and baseline PGI-S, were not significantly associated (Figure 1D,E). These findings suggest that younger participants were more likely to report treatment improvement at week 8.

### 3.2. Secondary Outcomes

Hair loss severity (PGI-S) showed a significant median reduction of −1.0 point from baseline to week 8 (*p* = 0.0008), with a response rate of 59.4% (19/32). In the subgroup with more severe hair loss at baseline (PGI-S ≥ 3), the response rate was 81.2%, with a median reduction of −1.5 points (*p* = 0.0011, Figure 2A). The 60 s hair count showed a response rate of 68.8% (22/32), with a median reduction of −1.5 hairs shed (*p* = 0.0048, Figure 2B). Post hoc subgroup analyses suggested greater reductions in young females with noticeable hair loss (n = 9; response rate 88.9%, median reduction −4.00, *p* = 0.0078) and in females with noticeable hair loss (n = 14; response rate 85.7%, median reduction −4.17, *p* = 0.0019; Table S1). These findings should be interpreted as hypothesis-generating.

Depressive symptoms (PHQ-9) showed a response rate of 65.6% (21/32) at week 8, with a median reduction of −1.0 point (*p* = 0.002, Figure 2C). In the subgroup with baseline PHQ-9 ≥ 5 (n = 14), the response rate was 85.7% (12/14), with a median reduction of −3.0 points (*p* = 0.0020; Table S2). Anxiety symptoms (GAD-7) and quality of life (WHOQOL-BREF) did not show a significant change at week 8 (Figure 2D).

Six adverse events were reported: gastroenteritis (n = 2), gastritis (n = 1), asthma (n = 1), coronavirus disease 2019 (COVID-19) (n = 1), and common cold (n = 1). All were assessed as unlikely to be related to the study product. No serious adverse events occurred, and no participant withdrew from the trial due to adverse events.

### 3.3. Gut Microbiome Composition Remained Stable After HQP08 Supplementation

Stool samples were collected at week 12 (4 weeks after the 8-week intervention ended) to assess post-treatment persistence of microbiota changes. As expected by design, no significant differences in species richness, diversity, or overall community composition (PERMANOVA, *p* = 0.456) were observed between baseline and week 12 (Figure 3A–D). Only a few species, such as *Merdimmobilis hominis*, showed nominal changes in LMMs adjusted for age, sex, and BMI, and none survived multiple testing correction (Figure 3C). Spearman correlation analyses identified several nominal associations between species-level changes and clinical outcomes, including *Bacteroides eggerthii* with PGI-C response (rho = −0.537, *p* = 0.0015) and *Bacteroides caccae* with ΔPHQ-9 (rho = 0.488, *p* = 0.0046). However, no association remained significant after FDR correction (Figure 3E).

### 3.4. Functional Pathway Changes Were Associated with Depressive Symptom Improvement

Mirroring the stability of gut microbiome composition, no pathway showed significant longitudinal changes among all participants after FDR correction (Figure 4A,B). In contrast, pathway-level changes were significantly associated with clinical improvements. Spearman correlation analyses revealed that changes in multiple pathways were significantly correlated with ΔPHQ-9 after FDR correction, including pathways involved in molybdopterin biosynthesis, purine ribonucleoside degradation, lipopolysaccharide biosynthesis, thiamine metabolism, glycogen biosynthesis, and adenosylcobalamin salvage (Figure 4C). Pathway associations with PGI-C, ΔPGI-S, and hair count were not significant after correction. These findings suggest that functional pathway changes were more closely linked to depressive symptom improvement than to hair-specific endpoints in this exploratory cohort.

### 3.5. B. longum HK003-like Profiles Were Detected in a Subset of Participants After Supplementation

Regarding the relative abundance of *B. longum*, the probiotic species contained in HQP08 showed an increasing trend from baseline to week 12 (median CLR: 0.558 to 1.12), but this change was not statistically significant (*p* = 0.236, Figure 5A). We therefore examined whether *B. longum* HK003-like strains could be detected at the strain level.

At the strain level, *B. longum* HK003-like profiles were detected in four participants (12.5%) at week 12, with no baseline detection, meeting our operational definition of engraftment (Figure 5B,C). Three of these four had no detectable *B. longum* at baseline, and only one showed replacement of an endogenous strain. Among participants with detectable endogenous *B. longum* at both time points, the majority (21/27) retained their original strain. Thus, engraftment occurred predominantly in the absence of baseline *B. longum*, whereas replacement of established strains was rare. Clinical changes in the four participants with *B. longum* HK003-like profiles were heterogeneous relative to the rest of the cohort and are presented descriptively in Figure S2. In the four participants with *B. longum* HK003-like profiles, numerical shifts were observed in pathways related to fatty acid biosynthesis, carbohydrate metabolism, and the Bifidobacterium shunt, but these were descriptive only (Figure S3A). Species-level contributor analysis could not distinguish *B. longum* HK003 from endogenous *B. longum* strains (Figure S3B).

## 4. Discussion

This pilot study provides initial human data on HQP08 supplementation in adults with self-reported hair loss. HQP08 supplementation was associated with within-participant changes in participant-reported hair-loss severity, self-counted hair shedding, and PHQ-9 scores, with no serious adverse events observed. Importantly, this study evaluated a probiotic strain previously shown to reverse alopecia-like phenotypes in mice [11], now examined in humans, with feasibility, safety, within-participant clinical changes, and exploratory strain-level readouts. To our knowledge, this is the first interventional study of an HK003-containing synbiotic in a hair loss population. These findings align with epidemiological evidence linking microbial imbalance to hair disorders via the gut–skin axis [26,27,28] and provide encouraging proof-of-concept support for HQP08 as a microbiome-targeted approach to hair loss management.

These preliminary data offer several exploratory insights that may inform future translational research. First, younger age was associated with higher odds of PGI-C response in univariable analysis. Second, strain-level metagenomics revealed engraftment of HK003-like strains in a subset of participants, consistent with detection of an HK003-like profile after supplementation in a minority of participants, predominantly those without baseline *B. longum*. Third, PHQ-9 scores decreased in two-thirds of participants, and functional pathway analyses identified associations between mood improvement and microbial metabolism of thiamine, vitamin B12, and lipopolysaccharide biosynthesis. While the modest reduction in a population with generally low baseline scores should not be interpreted as an established antidepressant effect, these findings represent a signal of potential benefit and generate hypotheses regarding the psychobiotic potential of Bifidobacterium species and the gut–brain–skin axis.

Following HQP08 supplementation, the overall structure of the gut microbiota remained unchanged, consistent with observations that probiotic supplementation often does not induce lasting community-level shifts in the healthy gut [29,30,31]. Importantly, we collected stool samples 4 weeks after supplementation ended, aiming to detect strains that persisted beyond the active dosing window and providing a more conservative test of engraftment. Despite community-level stability, HK003-like profile *B.longum* was detected in four participants at week 12. Three of these four had no detectable *B. longum* at baseline, whereas most participants with endogenous *B. longum* retained their original strains. This is consistent with previous human studies showing that engraftment is highly individualized and depends on baseline microbiome configuration [32]. This pattern aligns with ecological principles of colonization resistance and niche occupancy: exogenous strains are more likely to engraft when a compatible niche is vacant but may fail to replace resident strains already adapted to the host environment [31,32,33]. Clinical changes among the four participants with HK003-like profiles were heterogeneous, and given the very small number, these observations cannot support a strain–efficacy relationship (Figure S2). The absence of replacement among most participants with endogenous *B. longum* may reflect niche exclusion, substrate competition, or host-specific adaptation [33,34].

Beyond strain-level engraftment, we observed correlations between functional pathway changes and depressive symptom improvement (ΔPHQ-9). Pathways related to thiamine metabolism, adenosylcobalamin (vitamin B12) salvage, purine ribonucleoside degradation, and lipopolysaccharide (LPS) biosynthesis were significantly associated with ΔPHQ-9. *Bifidobacterium* species, including *B. longum*, are recognized for their capacity to synthesize essential B vitamins, which are critical for neurotransmitter regulation and mood stabilization [35,36,37]. LPS-induced systemic inflammation is a well-established driver of both depressive symptoms and disruption of hair follicle immune privilege [38,39]. Previous trials have demonstrated the “psychobiotic” potential of other *B. longum* strains in reducing depression scores [40,41,42]. In participants with *B. longum* HK003-like profiles, descriptive signals in carbohydrate metabolism and the *Bifidobacterium* shunt were observed, generating testable hypotheses for future studies. Together, these findings are consistent with the hypothesis that systemic effects on psychosocial stress and inflammation may contribute to the observed clinical changes in HQP08, with the gut–brain–skin axis remaining a framework for future investigation.

Several important limitations merit careful consideration, reflecting the preliminary, exploratory, and translational nature of this pilot investigation. First, the open-label, single-arm design and modest sample size (n = 32) preclude causal inference and increase susceptibility to expectancy, placebo, and regression-to-the-mean effects. Dermatologist-confirmed diagnosis, alopecia subtype, duration, pattern, and probable etiology were not systematically collected, and objective follicular assessments were not performed—the 60 s hair count measures shedding only, and the eight-week duration was insufficient to assess sustained hair growth. Second, stool samples were collected only at baseline and four weeks after the intervention ended, precluding capture of on-treatment microbial dynamics; temporal precedence therefore cannot be established, and pathway changes cannot be inferred to have mediated the week-8 clinical outcomes. Third, the HK003-like classification was informed by the same dataset used for detection, and only four participants showed HK003-like profiles, limiting strain–efficacy inference. Although corroborated by an independent marker-SNP criterion, these findings remain exploratory. Fourth, although subjects were asked to maintain their usual dietary pattern during the trial period, detailed diet information was not recorded. Despite these constraints, the study has notable strengths: a prospective design, integration of both patient-reported and objective hair-shedding outcomes, a favorable safety profile, and incorporation of metagenomic and strain-level analyses. These features support the feasibility of, and provide a hypothesis-generating foundation for, longer and larger controlled studies of HQP08 in hair loss populations.

## 5. Conclusions

In summary, this open-label pilot study found that HQP08 was well tolerated and was associated with within-participant changes in hair-loss measures and PHQ-9 scores. Exploratory detection of HK003-like profiles and associations between functional pathways and PHQ-9 changes generate hypotheses for future research. The integration of clinical outcomes with microbiome analyses provides a foundation for rigorous evaluation of HQP08 in adequately powered, randomized, double-blind, placebo-controlled trials.

## Figures and Tables

**Figure 1:**
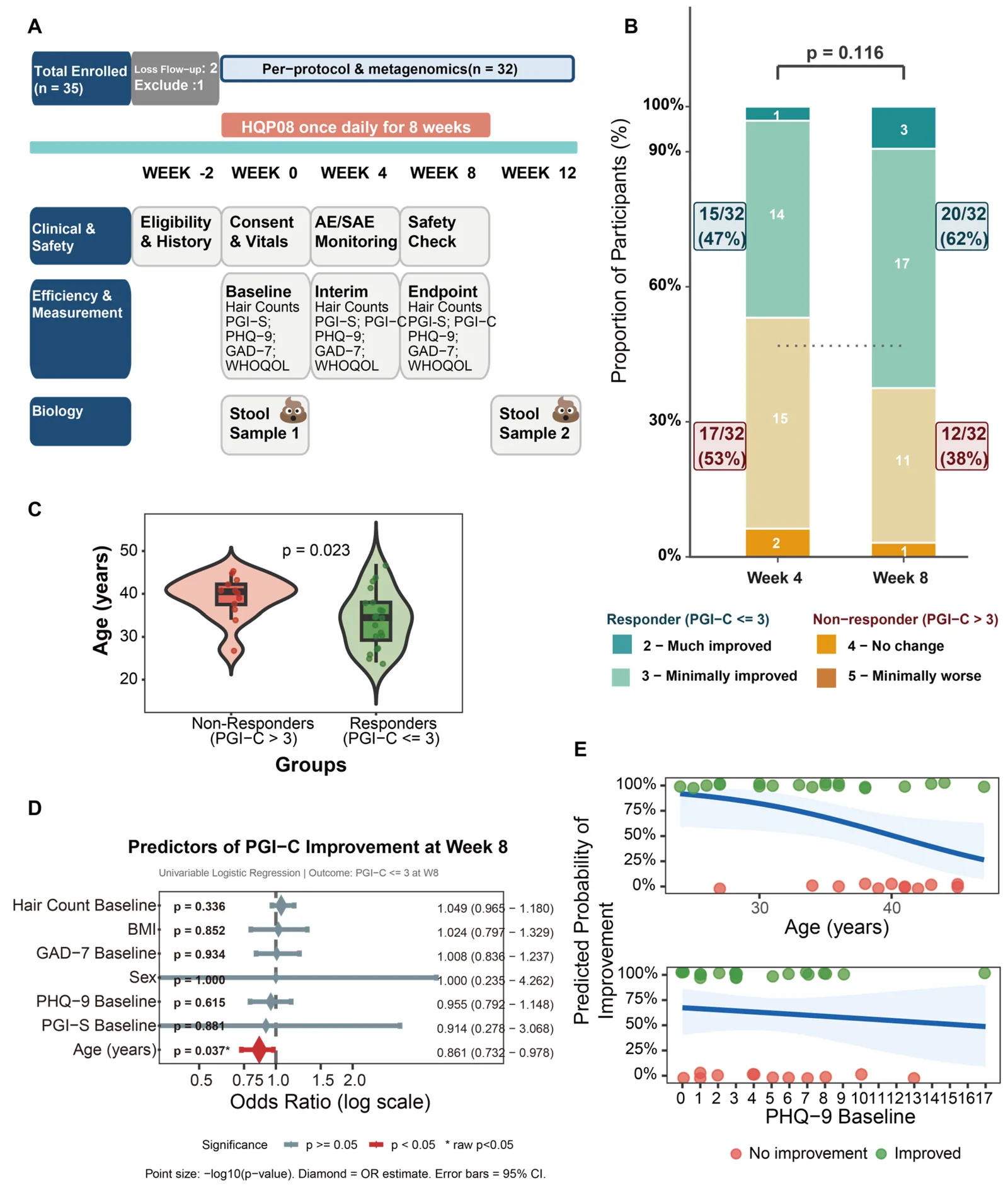
Study design and clinical outcomes following HQP08 supplementation. (**A**) Overview of participant flow and the open-label, single-arm pilot study design. (**B**) Distribution of PGI-C responses after supplementation. (**C**) Violin plot showing the age distribution in each group, with embedded boxplots indicating the median and interquartile range and individual points representing participants. Statistical significance was assessed using the Wilcoxon rank-sum test. (Responders: PGI-C ≤ 3; non-responders: PGI-C > 3) (**D**) Forest plot of odds ratios from univariable logistic regression analyses for each baseline characteristic. An odds ratio below 1 indicates that a higher value was associated with reduced odds of improvement. Points represent odds ratios and horizontal lines represent 95% confidence intervals; significant associations (*p* < 0.05) are highlighted in red. (**E**) Univariable logistic regression analysis showing the predicted probability of PGI-C improvement according to age and PHQ-9. Top: Age; Bottom: PHQ-9. Each point represents an individual participant and is colored by response status. The fitted logistic regression curve demonstrates a decreasing probability of clinical improvement with increasing value.

**Figure 2:**
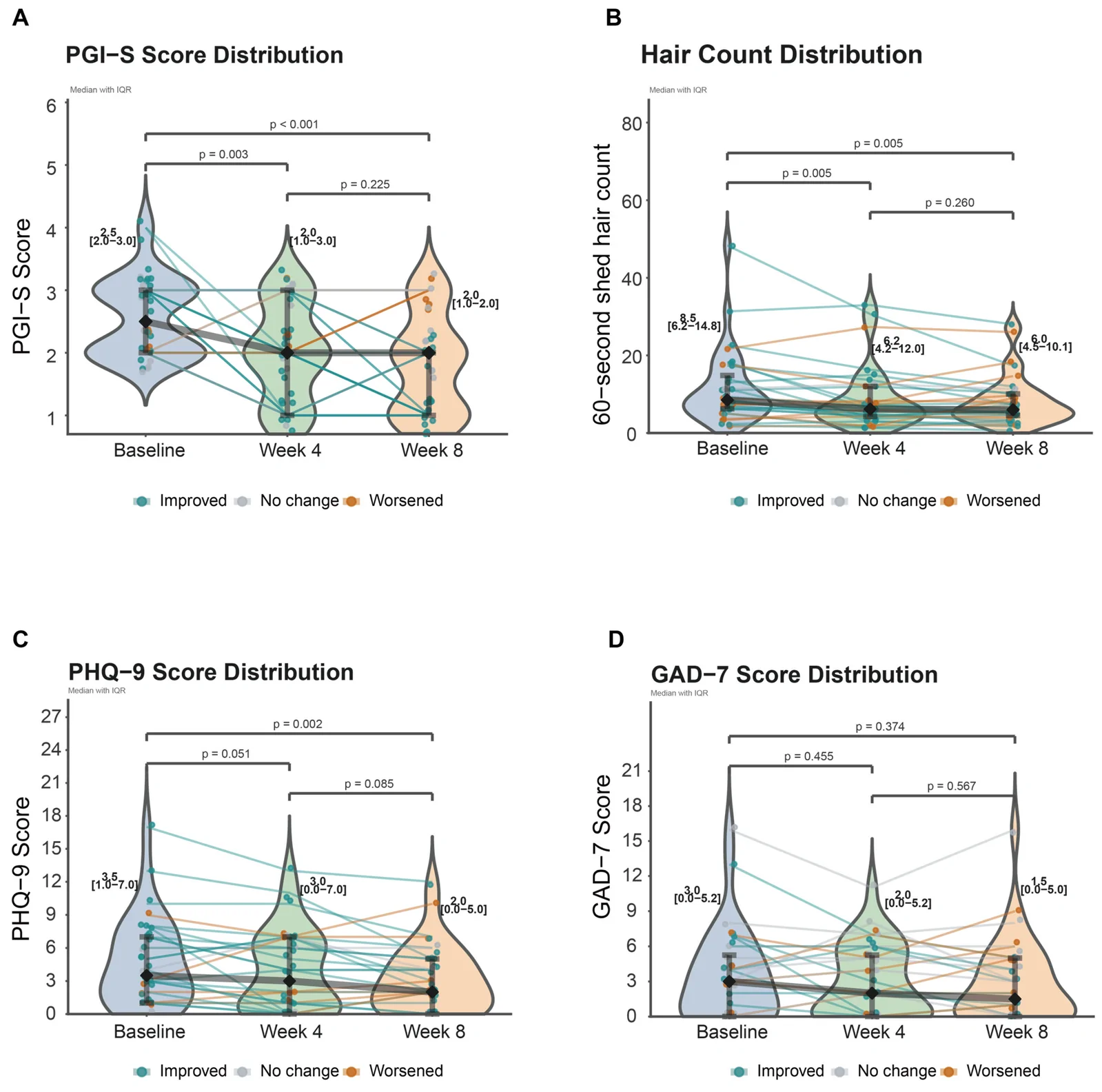
Secondary clinical outcomes after HQP08 supplementation. (**A**) Individual longitudinal changes in PGI-S hair loss severity scores following HQP08 supplementation. PGI-S scores range from 1 to 5 (none to very severe); lower scores indicate better outcomes. (**B**) Individual longitudinal changes in 60 s hair count across the intervention period. Lower count indicates reduced shedding. (**C**) Paired distribution of PHQ-9 depression scores at baseline, week 4 and week 8 in the overall cohort. Lower score indicates fewer depressive symptoms. (**D**) Paired distribution of GAD-7 anxiety scores at baseline, week 4 and week 8 in the overall cohort. Lower score indicates fewer anxiety symptoms.

**Figure 3:**
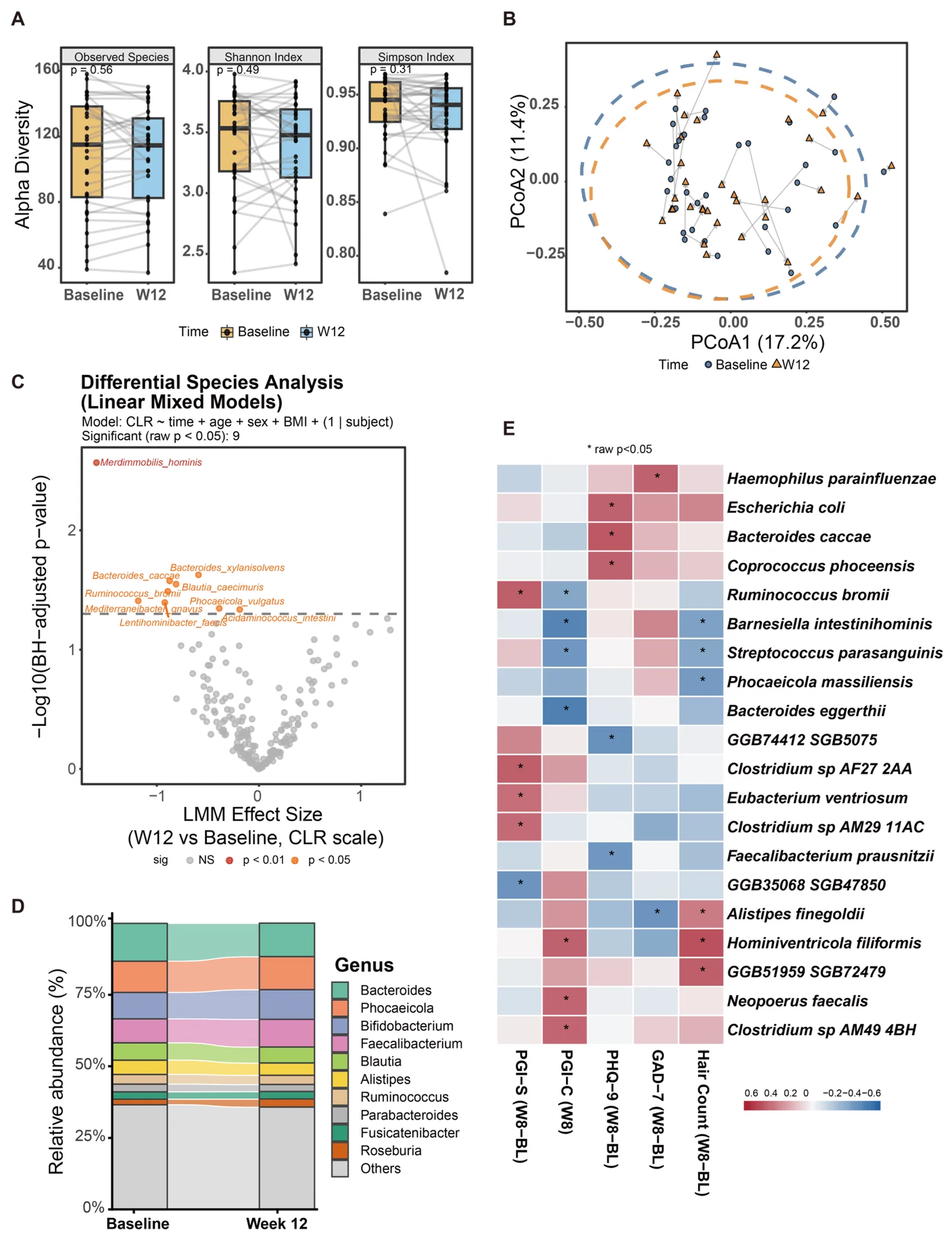
Gut microbiome profiles showed limited community-level variation following HQP08 supplementation. (**A**) Alpha diversity metrics at baseline and week 12. (**B**) PCoA analysis based on Bray–Curtis dissimilarity. Paired PERMANOVA: R^2^ = 0.005, F = 0.313, *p* = 0.456 | Beta dispersion *p* = 0.8662. Dashed circles refer to 95% data ellipses for each time point. (**C**) Volcano plot depicting significantly altered species identified by linear mixed models (adjusted for age, sex, baseline BMI), with nominal significance (*p* < 0.05). (**D**) Bar chart demonstrating differences in genus-level bacterial abundance between baseline and week 12. (**E**) Spearman correlation heatmap showing exploratory associations between species-level microbial changes and changes in clinical outcomes.

**Figure 4:**
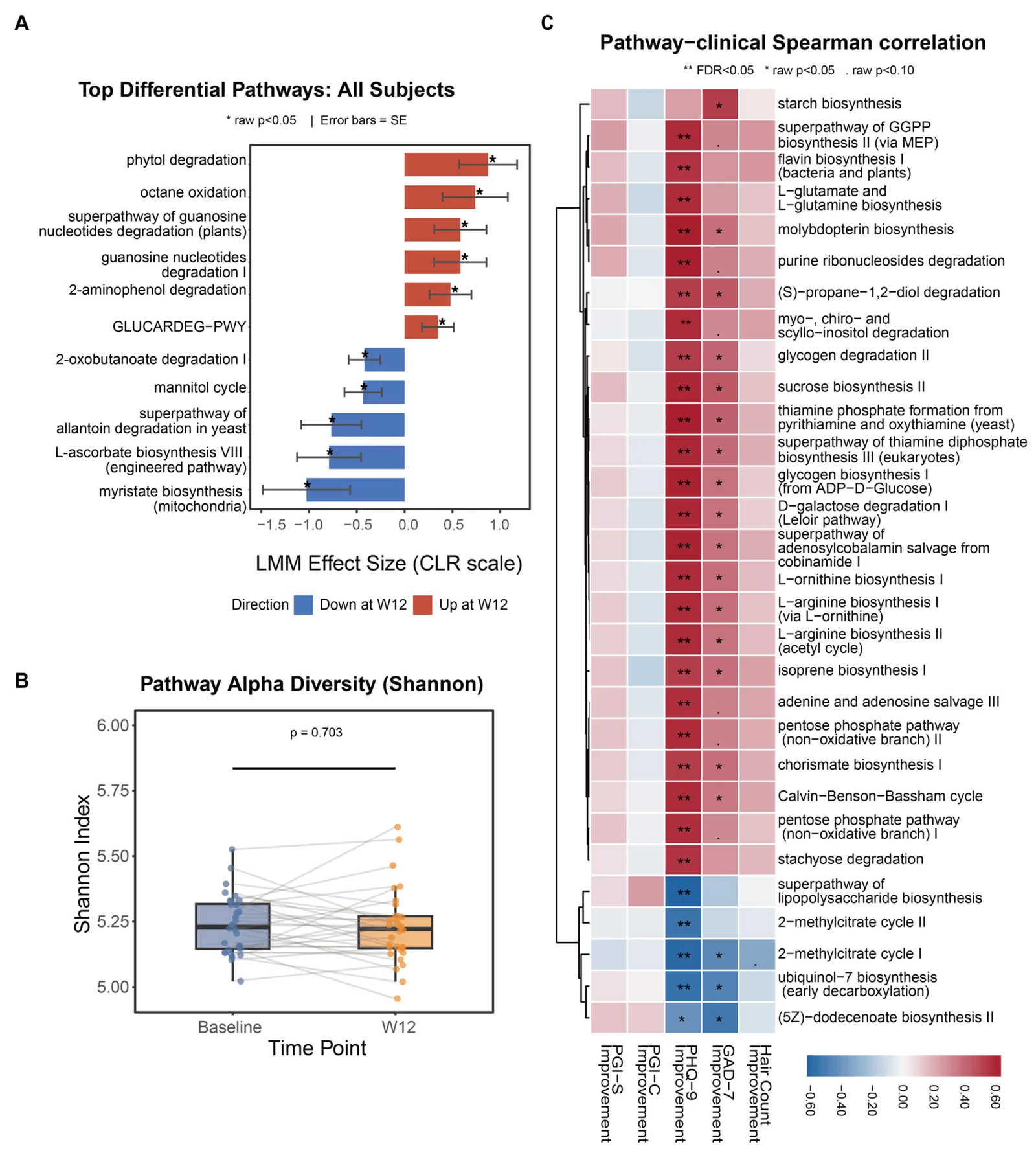
Longitudinal microbiome functional changes following HQP08 supplementation. (**A**) Bar plots showing significantly different pathway abundances between baseline and week 12 in the full cohort. Pathway abundances were derived from HUMAnN3 profiling and analyzed using linear mixed-effects models adjusted for age, sex, and BMI, with subject-level random intercepts. (**B**) Alpha diversity (Shannon index) of functional pathways at baseline and week 12. (**C**) Associations between longitudinal pathway changes and clinical improvement. Heatmap showing improvement-oriented Spearman correlations between longitudinal changes in CLR-transformed MetaCyc pathway abundance and changes in clinical outcomes, including PGI-S, PGI-C, PHQ-9, GAD-7, and hair count. Since lower values indicate better outcomes for all clinical variables, the displayed correlation coefficient was calculated as improvement-oriented ρ = −Spearman’s ρ. Red indicates that greater pathway increases were associated with better clinical improvement, whereas blue indicates association with poorer improvement. Pathway rows are displayed using mapped MetaCyc pathway names. Significance labels indicate ** FDR < 0.05, * nominal *p* < 0.05.

**Figure 5:**
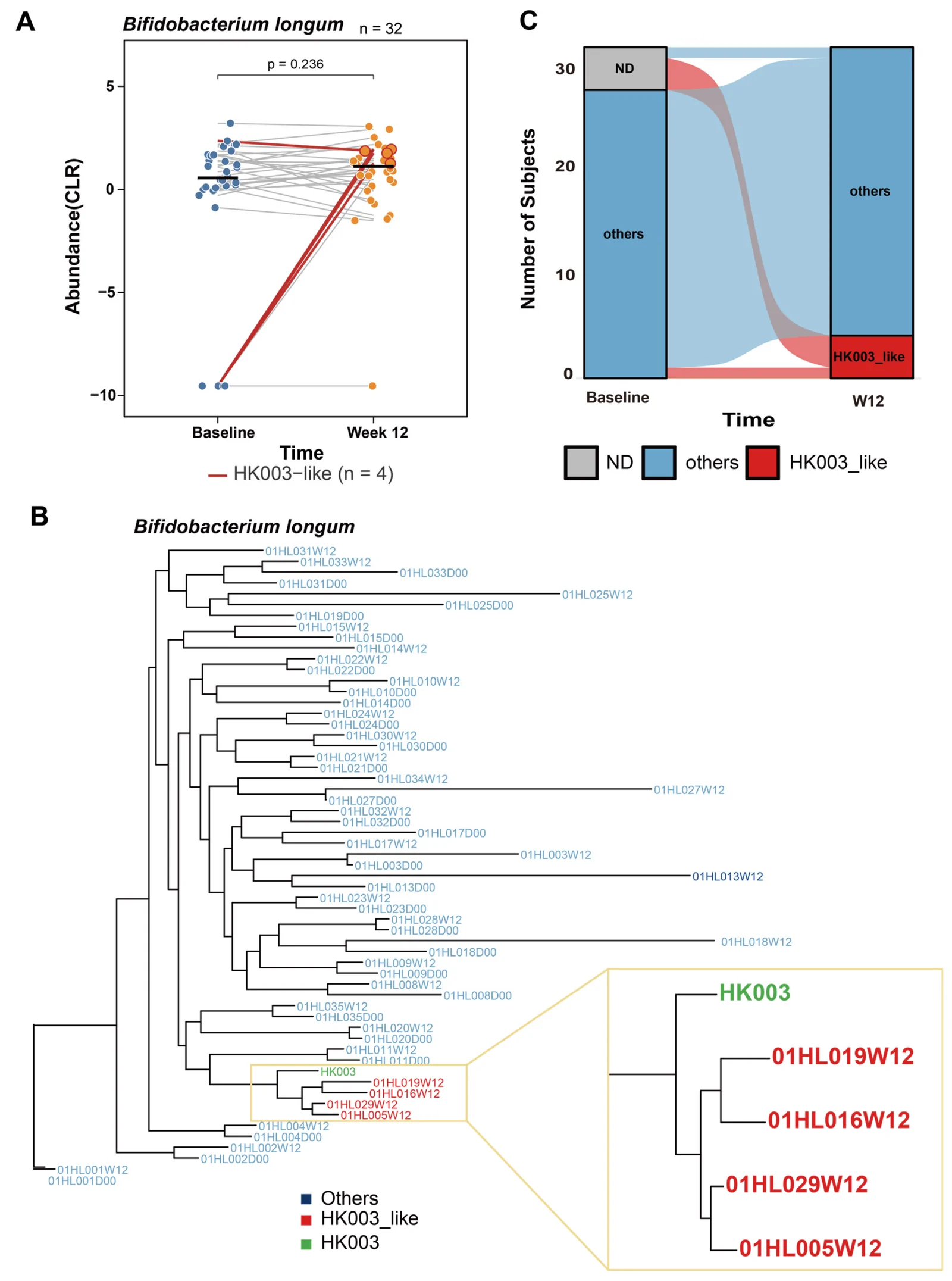
Strain-resolved metagenomic analysis reveals heterogeneous engraftment of the supplemented *Bifidobacterium longum* HK003 strain. (**A**) Paired changes in the relative abundance of *Bifidobacterium longum.* Left, blue: Baseline; Right, yellow: Week12. Red line: HK003-like samples. (**B**) Phylogenetic tree of *B. longum* strains reconstructed from metagenomic marker genes. Samples from baseline and week 12 were included together with the supplemented reference strain HK003 (green text). (**C**) Alluvial plot showing subject-level *B. longum* strain cluster transitions from baseline to week 12. Each stream represents participants moving between strain cluster states over time, including endogenous strain clusters, not-detected status (gray), and HK003-like strain status (red).

**Table 1:** Baseline Characteristics of Study Participants.

Characteristics	
Age, years	36.0 ± 6.5
Sex	
Male	16 (50.0)
Female	16 (50.0)
Self-reported hair loss severity (PGI-S)	
Mild	16 (50.0)
Moderate	14 (43.8)
Severe	2 (6.2)
60 s hair count	8.5 (6.2–14.8)
PHQ-9 depression score	3.5 (1.0–7.0)
PHQ-9 severity category	
Minimal (0–4)	18 (56.2)
Mild (5–9)	11 (34.4)
Moderate (10–14)	2 (6.2)
Moderately severe (15–19)	1 (3.1)
GAD-7 anxiety score	3.0 (0–5.3)
GAD-7 severity category	
Minimal (0–4)	23 (71.9)
Mild (5–9)	7 (21.9)
Moderate (10–14)	1 (3.1)
Severe (15–21)	1 (3.1)
Quality of Life (WHOQOL-BREF)	
Physical health domain	56.0 (50.0–63.0)
Psychological domain	53.0 (44.0–63.0)
Social relationships domain	75.0 (54.5–76.5)
Environment domain	63.0 (56.0–69.0)
Smoking status	
Non-smoker	28 (87.5)
Current smoker	2 (6.2)
Ex-smoker	2 (6.2)
Alcohol consumption	
Never	15 (46.9)
Social drinker	17 (53.1)
Body Mass Index (BMI), kg/m^2^	22.8 ± 2.9
Co-morbidities	3 (9.4)
Hypothyroidism	1 (3.1)
Rheumatism	1 (3.1)
Hepatitis B	1 (3.1)

Data are presented as mean ± SD, median (IQR), or *n* (%), as appropriate. BMI, body mass index; GAD-7, Generalized Anxiety Disorder-7; PGI-S, Patient Global Impression of Severity; PHQ-9, Patient Health Questionnaire-9; WHOQOL-BREF, World Health Organization Quality of Life-Brief Version.

## Data Availability

The metagenomic sequencing data generated in this study have been deposited in the National Library of Medicine (NCBI) under accession code PRJNA1471447. De-identified clinical metadata is available upon appropriate request to the corresponding author.

## Supplementary Materials

The following supporting information can be downloaded at https://www.mdpi.com/article/10.3390/nu18193111/s1, Figure S1: PGI-C response distributions and sensitivity analysis of the responder definition; Figure S2: Clinical profiles of the four participants with *B. longum* HK003-like profiles at week 12; Figure S3: Functional pathway changes and species-level contributions in the participants with *B. longum* HK003-like profiles; Table S1: Post-hoc subgroup analysis of 60-s hair count improvement at week 8; Table S2: Proportion of improvement in individual items of PHQ-9 at week 8 (PHQ-9 ≥ 5 sub-group, n = 14).

## Abbreviations

The following abbreviations are used in this manuscript:

PGI-C	Patient Global Impression of Change
PGI-S	Patient Global Impression of Severity
PHQ-9	Patient Health Questionnaire-9
GAD-7	Generalized Anxiety Disorder-7
WHOQOL-BREF	World Health Organization Quality of Life-Brief Version
FOS	Fructooligosaccharides
GOS	Galactooligosaccharides
LPS	Lipopolysaccharide
FMT	fecal microbiota transplantation
BMI	body mass index
LMMs	linear mixed models
CLR	centered-log-ratio transformation
PCoA	principal coordinate analysis
