# Supplementary material for: Synbiotic HQP08 Supplementation for Hair Loss and Depressive Symptoms: An Open-Label Pilot Study of Clinical Outcomes and Gut Microbiome Changes
Source: Nutrients. 2026 Sep 22;18(19):3111. doi: 10.3390/nu18193111 (PMC13666846; doi:10.3390/nu18193111)
Supplement: Supplementary file 1 [file nutrients-18-03111-s001.zip › figs_revised_final_nutritents_Supplementary/Fig_S1.pdf]

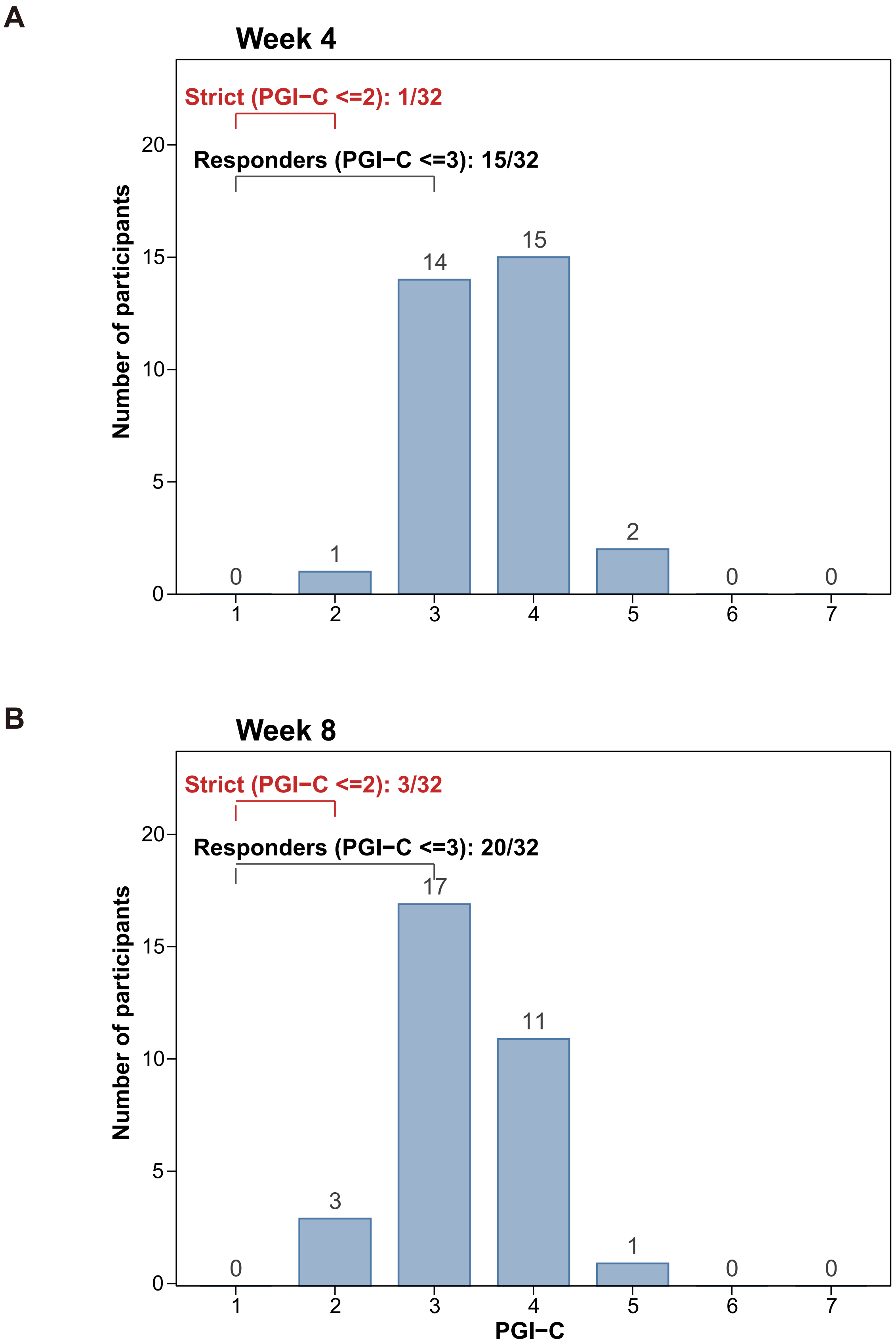
A
Week 4
Strict (PGI−C <=2): 1/32
Responders (PGI−C <=3): 15/32
Number of participants
0
5
10
15
20
0
1
14
15
2
0
0
1
2
3
4
5
6
7
B
Week 8
Strict (PGI−C <=2): 3/32
Responders (PGI−C <=3): 20/32
Number of participants
0
5
10
15
20
0
3
17
11
1
0
0
1
2
3
4
5
6
7
PGI−C
