# Supplementary material for: Synbiotic HQP08 Supplementation for Hair Loss and Depressive Symptoms: An Open-Label Pilot Study of Clinical Outcomes and Gut Microbiome Changes
Source: Nutrients. 2026 Sep 22;18(19):3111. doi: 10.3390/nu18193111 (PMC13666846; doi:10.3390/nu18193111)
Supplement: Supplementary file 1 [file nutrients-18-03111-s001.zip › figs_revised_final_nutritents_Supplementary/Fig_S2.pdf]

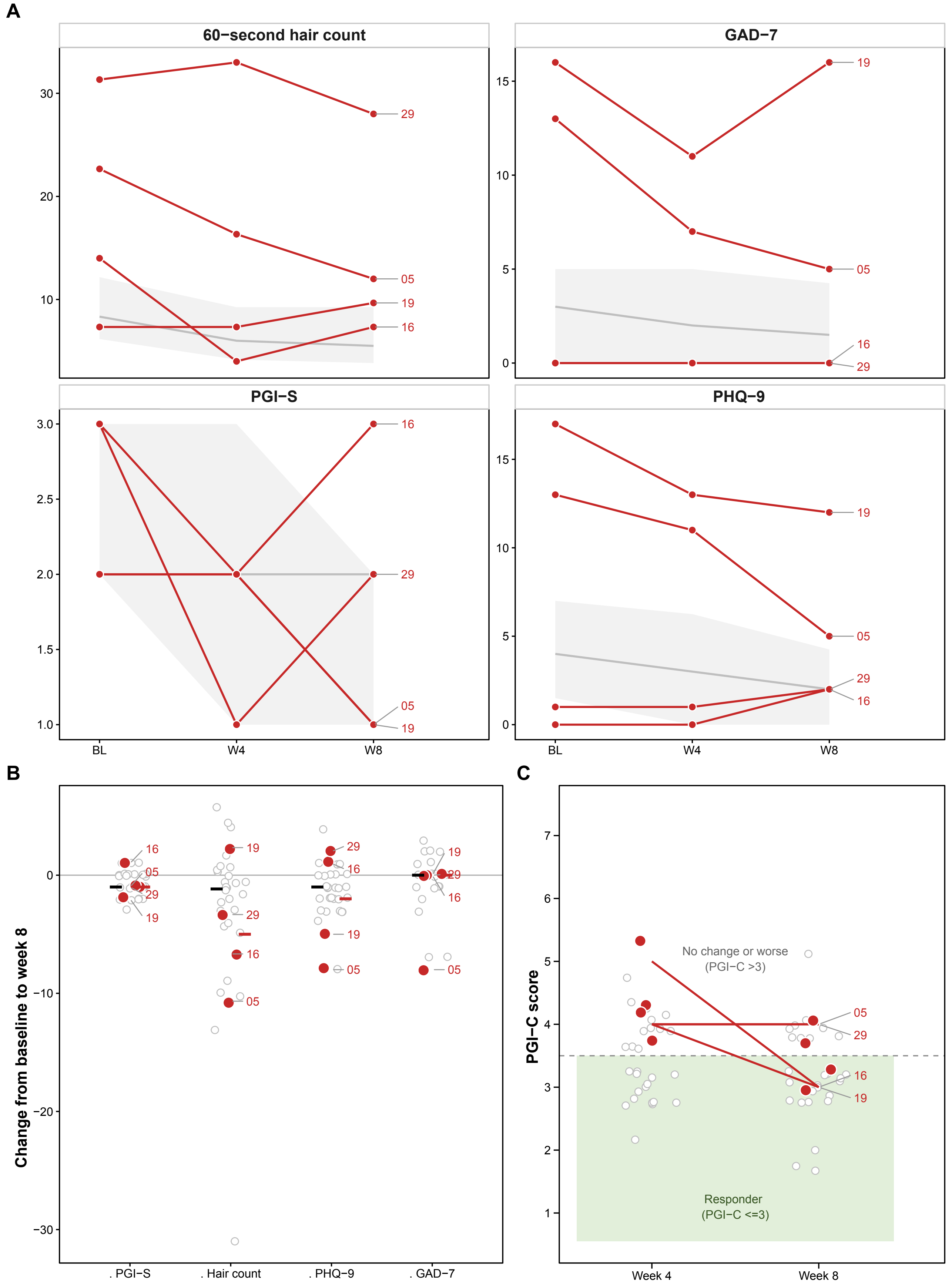
A
60−second hair count
GAD−7
PGI−S
PHQ−9
BL
W4
W8
29
05
19
16
B
C
Change from baseline to week 8
. PGI−S
. Hair count
. PHQ−9
. GAD−7
PGI−C score
No change or worse
(PGI−C >3)
Responder
(PGI−C <=3)
Week 4
Week 8
