# Supplementary material for: Synbiotic HQP08 Supplementation for Hair Loss and Depressive Symptoms: An Open-Label Pilot Study of Clinical Outcomes and Gut Microbiome Changes
Source: Nutrients. 2026 Sep 22;18(19):3111. doi: 10.3390/nu18193111 (PMC13666846; doi:10.3390/nu18193111)
Supplement: Supplementary file 1 [file nutrients-18-03111-s001.zip › figs_revised_final_nutritents_Supplementary/Fig_S3.pdf]

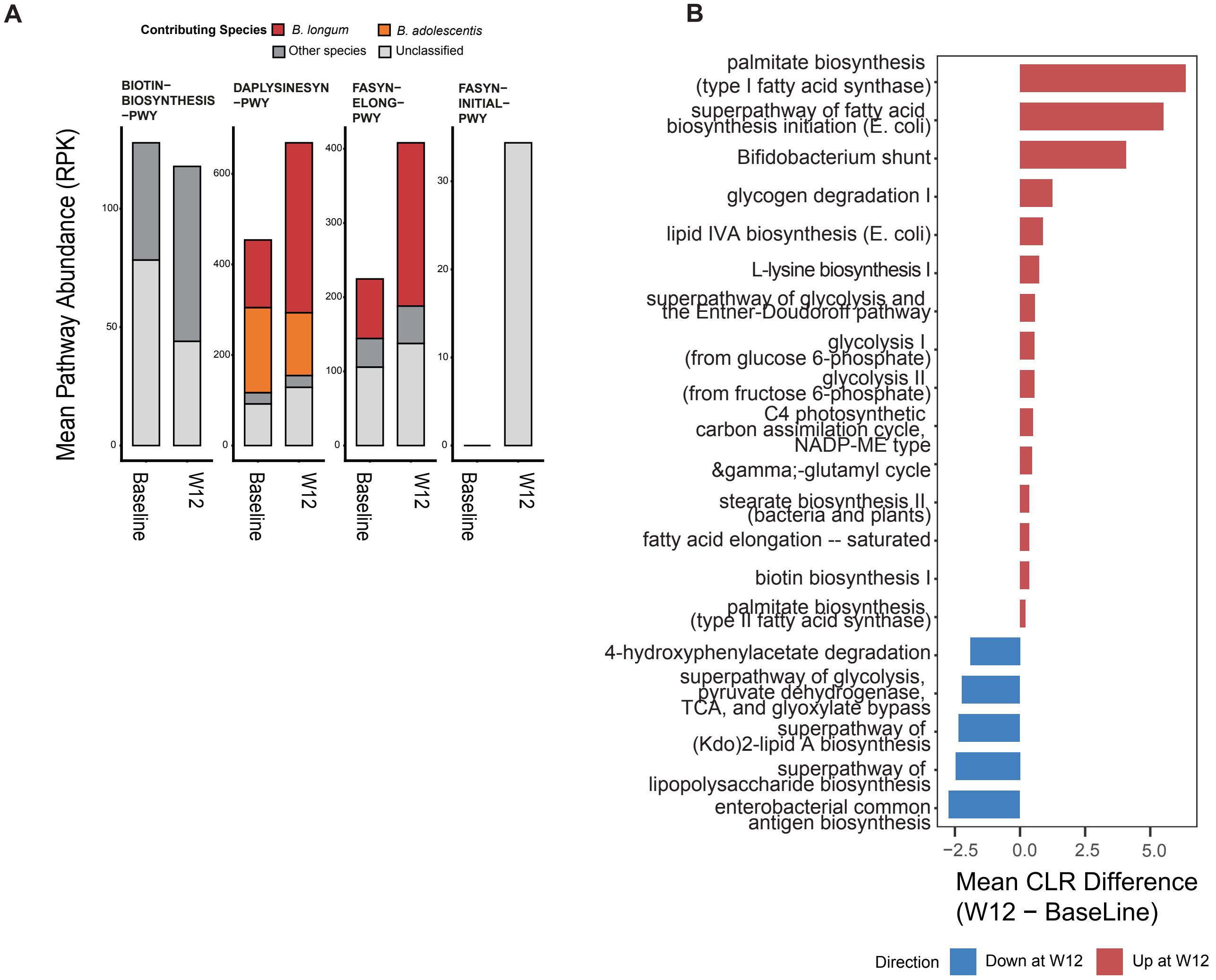
A
Contributing Species
B. longum
B. adolescentis
Other species
Unclassified
BIOTIN−BIOSYNTHESIS−PWY
DAPLYSINESYN−PWY
FASYN−ELONG−PWY
FASYN−INITIAL−PWY
Mean Pathway Abundance (RPK)
0
50
100
200
400
600
300
10
20
30
Baseline
W12
B
palmitate biosynthesis (type I fatty acid synthase)
superpathway of fatty acid biosynthesis initiation (E. coli)
Bifidobacterium shunt
glycogen degradation I
lipid IVA biosynthesis (E. coli)
L-lysine biosynthesis I
superpathway of glycolysis and the Entner-Doudoroff pathway
glycolysis I (from glucose 6-phosphate)
glycolysis II (from fructose 6-phosphate)
C4 photosynthetic carbon assimilation cycle, NADP-ME type
γ-glutamyl cycle
stearate biosynthesis II (bacteria and plants)
fatty acid elongation -- saturated
biotin biosynthesis I
palmitate biosynthesis (type II fatty acid synthase)
4-hydroxyphenylacetate degradation
superpathway of glycolysis, pyruvate dehydrogenase, TCA, and glyoxylate bypass
superpathway of (Kdo)2-lipid A biosynthesis
superpathway of lipopolysaccharide biosynthesis
enterobacterial common antigen biosynthesis
−2.5
0.0
2.5
5.0
Mean CLR Difference (W12 − BaseLine)
Direction
Down at W12
Up at W12
