# Supplementary material for: Synbiotic HQP08 Supplementation for Hair Loss and Depressive Symptoms: An Open-Label Pilot Study of Clinical Outcomes and Gut Microbiome Changes
Source: Nutrients. 2026 Sep 22;18(19):3111. doi: 10.3390/nu18193111 (PMC13666846; doi:10.3390/nu18193111)
Supplement: Supplementary file 1 [file nutrients-18-03111-s001.zip › nutrients-4516788-tables S1 and S2.pdf]

Supplementary

Table S1: Post-hoc subgroup analysis of 60-second hair count improvement at week 8

Subgroup	Response rate	Median change in hair shed	p-value
Young female (<40 years) with noticeable hair loss (≥5 hairs shed) (n=9)	88.9%	-4.00	p=0.0078
Female with noticeable hair loss (≥5 hairs shed) (n=14)	85.7%	-4.17	p=0.00187
Young adults (<40 years) with noticeable hair loss (≥5 hairs shed) (n=18)	83.3%	-2.17	p=0.0056
Young adults (<40 years) (n=21)	81.0%	-1.67	p=0.0025
Young male (<40 years) with noticeable hair loss (≥5 hairs shed) (n=9)	77.8%	-1.67	p=0.3594
Noticeable hair loss (≥5 hairs shed) (n=27)	74.1%	-2.33	p=0.0048
Male with noticeable hair loss (≥5 hairs shed) (n=13)	61.5%	-1.00	p=0.7354

Table S2: Proportion of improvement in individual items of PHQ-9 at week 8 (PHQ-9 $\geq$ 5 subgroup, n=14)

Item	Number of patients (Percentage); n=14
Item 1: Little interest or pleasure in doing things	5 (35.7)
Item 2: Feeling down, depressed or hopeless	7 (50.0)
Item 3: Trouble falling asleep, staying asleep, or sleeping too much	5 (35.7)
Item 4: Feeling tired or having little energy	5 (35.7)
Item 5: Poor appetite or overeating	7 (50.0)
Item 6: Feeling bad about yourself - or that you're a failure or have let yourself or your family down	8 (57.1)
Item 7: Trouble concentrating on things, such as reading the newspaper or watching television	4 (28.6)
Item 8: Moving or speaking so slowly that other people could have noticed. Or, the opposite - being so fidgety or restless that you have been moving around a lot more than usual	4 (28.6)
Item 9: Thoughts that you would be better off dead or of hurting yourself in some way	1 (7.1)
